# Interactive Web Tool for Standardizing Proteomics Workflow for Liquid Chromatography-Mass Spectrometry Data

**Published:** 2019-05-23

**Authors:** Sudhir Srivastava, Michael Merchant, Anil Rai, Shesh N. Rai

**Affiliations:** 1 Centre for Agricultural Bioinformatics, ICAR-Indian Agricultural Statistics Research Institute, New Delhi, India; 2 Department of Bioinformatics & Biostatistics, University of Louisville, Louisville, Kentucky, United States of America; 3 Department of Medicine, University of Louisville, Louisville, Kentucky, United States of America; 4 Department of Pharmacology & Toxicology, University of Louisville, Louisville, Kentucky, United States of America; 5 Biostatistics and Bioinformatics Facility, James Graham Brown Cancer Center, University of Louisville, Louisville, Kentucky, United States of America

**Keywords:** Coefficient of variation, Imputation, Peptides, Proteins, Sum of squares, Technical variability

## Abstract

**Introduction:**

The proteomics experiments involve several steps and there are many choices available for each step in the workflow. Therefore, standardization of proteomics workflow is an essential task for design of proteomics experiments. However, there are challenges associated with the quantitative measurements based on liquid chromatography-mass spectrometry such as heterogeneity due to technical variability and missing values.

**Methods:**

We introduce a web application, Proteomics Workflow Standardization Tool (PWST) to standardize the proteomics workflow. The tool will be helpful in deciding the most suitable choice for each step of the experimentation. This is based on identifying steps/choices with least variability such as comparing Coefficient of Variation (CV). We demonstrate the tool on data with categorical and continuous variables. We have used the special cases of general linear model, analysis of covariance and analysis of variance with fixed effects to study the effects due to various sources of variability. We have provided various options that will aid in finding the contribution of sum of squares for each variable and the CV. The user can analyze the data variability at protein and peptide level even in the presence of missing values.

**Availability and implementation:**

The source code for “PWST” is written in R and implemented as shiny web application that can be accessed freely from https://ulbbf.shinyapps.io/pwst/.

## Introduction

Standardization of experimental workflow is an essential task for carrying out proteomics experiments [1,2]. There are various technical steps involved in proteomics experiments such as sample collection, sample storage, sample preparation, extraction, Liquid Chromatography (LC) separation and Mass Spectrometry (MS) detection. The experimenters have various choices available for each step in the proteomics workflow. Therefore, it becomes necessary to find the most suitable choice for each step in the proteomics workflow. LC-MS is used in proteomics as a method for identification and quantification of features (peptides/ proteins) in complex mixtures [3,4]. There are several challenges associated with the proteomics data such as data heterogeneity due to technical reasons, Missing Values (MVs) and low-abundant features. Furthermore, the proteomics data can be either balanced (equal number of observations in each group) or unbalanced (unequal number of observations in each group). The data can be unbalanced due to unequal number of subjects, or missing observations, or both. The MVs in proteomics data can occur due to biological and/or technical issues. The missing observations are broadly categorized as Missing Completely at Random (MCAR), Missing at Random (MAR) and Missing not at Random (MNAR) [5].

We have developed a user-friendly tool for standardizing the proteomics workflow and studying the variability in proteomic expression data generated by high throughput technologies involving MS [6–9]. We use the special cases of General Linear Model (GLM), analysis of covariance and analysis of variance to study the data variability. The user can estimate the contribution of various sources of variation to the overall variability. The study of data variability can be done using various analysis methods and normalization techniques. The user can analyze the data either by excluding the features having missing observations or by imputing the MVs. Excluding the features having missing observations leads to loss of information from the experiment. Therefore, we have provided two imputation methods to include more number of features in the analysis. We have demonstrated the tool using a simulated proteomics data comprising of 1000 peptides corresponding to 200 proteins. We implemented all the steps in R [10] and used “shiny” package [11] for developing the web application.

## Methods

The steps and various options available under each step are described below. Please see “Supplementary File 1” for more details about all the steps.

### Upload the expression data

The user has to upload the proteomics expression data. Please see “Supplementary File 1” for more details about the data format. We have provided an example proteomics data (Supplementary File 2).

### Feature type

The analysis can be done either at protein level or peptide level. After uploading the expression data file, the user has to select the feature type.

### Aggregation method

We have provided four options for data aggregation: (i) Mean, (ii) Median, (iii) Sum, (iv) Maximum. Data aggregation is required if the user has provided the peptide data and wants analysis at protein level. It is also applicable to other situations, such as when the features (proteins or peptides) are redundant. For example, if the user uses more than one database for searching features, there may be many redundant features.

### Upload the additional information

The user has to upload the additional information about the data. This file contains the information of the samples and the variables under study. The variables may be categorical and/or continuous (numeric). Please see “Supplementary File 1” for more details about the data format. We have provided an example additional information data (Supplementary File 2).

### Choose the categorical variables

The user has to select the categorical variables which will automatically pop out after the file containing additional information has been uploaded. Examples of the categorical variables in proteomics workflow are: storage methods, extraction methods, etc.

### Choose the numeric variables

After selecting the categorical variables, the user can now select the numeric (continuous) variable from the remaining variables, if available. Examples of numeric variables are age, weight, height, etc. of the individuals.

### Analysis method

We have provided two options for the analysis:

#### Excluding missing values:

Features having MVs in any of the samples are discarded from the analysis. The features having observations in all the samples are retained for analysis.

#### Imputing missing values:

The MVs are imputed after applying the normalization methods to the data [12] as given in next section. We have provided two imputation methods under the assumption of MAR or MCAR, namely, SVD [13] and KNN [14,15] available from the “impute. MAR” function of the R package “imputeLCMD” [16]. We impute the data at protein level if the data is available at protein level. Otherwise, we impute the data at peptide level. In case, if the analysis is to be done at protein level for the peptide data, then we first impute the data at peptide level and then aggregate the data. By default, the imputation is done globally. However, the user can apply the imputation methods group wise by specifying additional column “Norm_Imp_Group” and the group numbers in the file containing additional information.

### Transformation/Normalization method

There are four options available for data transformation and/or normalization:

#### Logarithmic transformation:

The raw data is transformed by taking log base 2.

#### Quantile Normalization (QN):

This method is applied on log base 2 transformed data using the “normalize.quantiles” method [17] available in R package “preprocessCore” [18].

#### Variance Stabilizing Normalization (VSN):

This method is applied on the raw data using “justvsn” function available in R package “vsn” [19].

#### None:

In some situations, if the user wants to use his own normalized data, then he can use the “None” option.

By default, the normalization methods (QN and VSN) are applied globally. The user can apply the normalization methods (QN and VSN) group wise by specifying additional column “Norm_Imp_Group” and the group numbers in the file containing additional information.

### Level of significance

The user can specify the level of significance (alpha). By default, the level of significance is 0.05.

### Method of adjustment

The user must adjust the p-values for multiple testing of features for which we have provided the following options: “BH”, “bonferroni”, “holm”, “Hochberg”, “hommel” and “BY” [10]. The method “BH” is the default adjustment method.

The user has to hit the “Submit” button after specifying the above mentioned inputs. The user will get the following results under different tabs:

#### Inputs selected:

It shows the various inputs defined by the user for the analysis.

#### Visual plots of the preprocessed data:

We provide exploratory plots of the preprocessed data such as box plot, density plot, correlation heatmap.

#### The Sum of Squares (SS) results:

We fit the ANOVA/ ANCOVA model with fixed effects for each feature. The results comprise of: (i) A table showing the contribution of SS due to each variable, the p-values and the adjusted p-values corresponding to each variable, (ii) summary of % contribution of SS and (iii) box plot showing % contribution of SS due to each variable.

#### The Coefficient of Variation (CV) analysis:

We calculate the CV corresponding to the groups within each categorical variable. The results consist of: (i) A table showing the CV of different groups of each categorical variable for all the features, (ii) summary of CV and (iii) box plot showing CV under the various groups of categorical variables.

#### Number of significant features:

We provided a table showing the number of features without and with adjustment which have significant effect due to each variable.

All these results can be viewed and downloaded. The results and their descriptions are given in “Supplementary File 1”.

## Demonstration

We used a simulated dataset for demonstrating our tool. We generated a proteomics expression data set that consists of 200 proteins with 1000 peptides. This simulated data mimics the data in recently published article [2]. Please see files “Supplementary Files 2 and 3” for proteomics expressions and additional information, respectively. In this data set, variability is due to two steps: Ml - tissue storage method, and M2 - tissue extraction method. Furthermore, step Ml has two levels (A1 & A2) and step M2 has three levels (Bl, B2 and B3), each with three biological replicates. Also, the MS procedure is repeated twice (two runs) with resulting sample size of 36 (2 × 3 × 3 × 2); the data structure is of a three-factor balanced ANOVA model. We have also included “Age” of the subjects (biological replicates) as continuous variable. Statistical analyses involve ANCOVA model. The purpose is to select the most suitable (less variability) levels in steps Ml and M2. The webtool can easily accommodate multiple steps (≥ 1) with multiple levels (≥ 2). We analyzed the data at protein level using VSN normalization and SVD imputation method. By providing various inputs to the tool, the user gets various results. Based on the summary and box plots, we found that the SS contribution due to the variable M2 is more than that of variable Ml. We found that the variable “Age” has the least SS contribution. Furthermore, the summary and box plots of CV show that (i) within variable Ml, A2 has lesser variability that of Al and (ii) within variable M2, B2 has the least variability among the three approaches of M2. Therefore, we can conclude that (i) approach A2 is better than that of Al for the method Ml, (ii) approach B2 is better than those of Bl and B3 for the method M2.

## Conclusion

Our tool provides a user-friendly approach to standardize proteomics workflow using multiple statistical approaches. The user can identify the steps with least variability based on SS and CV. The tool will be helpful to the researchers for designing and executing the experiments.

## Supplementary Material

Suppl 1 Details and demonstration of the tool

Suppl 2 An example of proteomics expression data

Suppl 3 An example of additional information of data

## List of Abbreviations:

LC: Liquid Chromatography
MS: Mass Spectrometry
GLM: General Linear Model
ANOVA: Analysis of Variance
ANCOVA: Analysis of Covariance
MCAR: Missing Completely at Random
MAR: Missing at Random
MNAR: Missing not at Random
MVs: Missing Values
SS: Sum of Squares
CV: Coefficient of Variation
PWST: Proteomics Workflow Standardization Tool
