# Supplementary material for: Interactive Web Tool for Standardizing Proteomics Workflow for Liquid Chromatography-Mass Spectrometry Data
Source: J Proteomics Bioinform. Author manuscript; Available in PMC 2020 Mar 6. (PMC7059686)
Supplement: Suppl 1 — Details and demonstration of the tool [file NIHMS1034721-supplement-Suppl_1.docx]

**Proteomics Workflow Standardization Tool**

The PWST tool can be accessed from https://ulbbf.shinyapps.io/pwst/. The screenshot of the tool is given below:


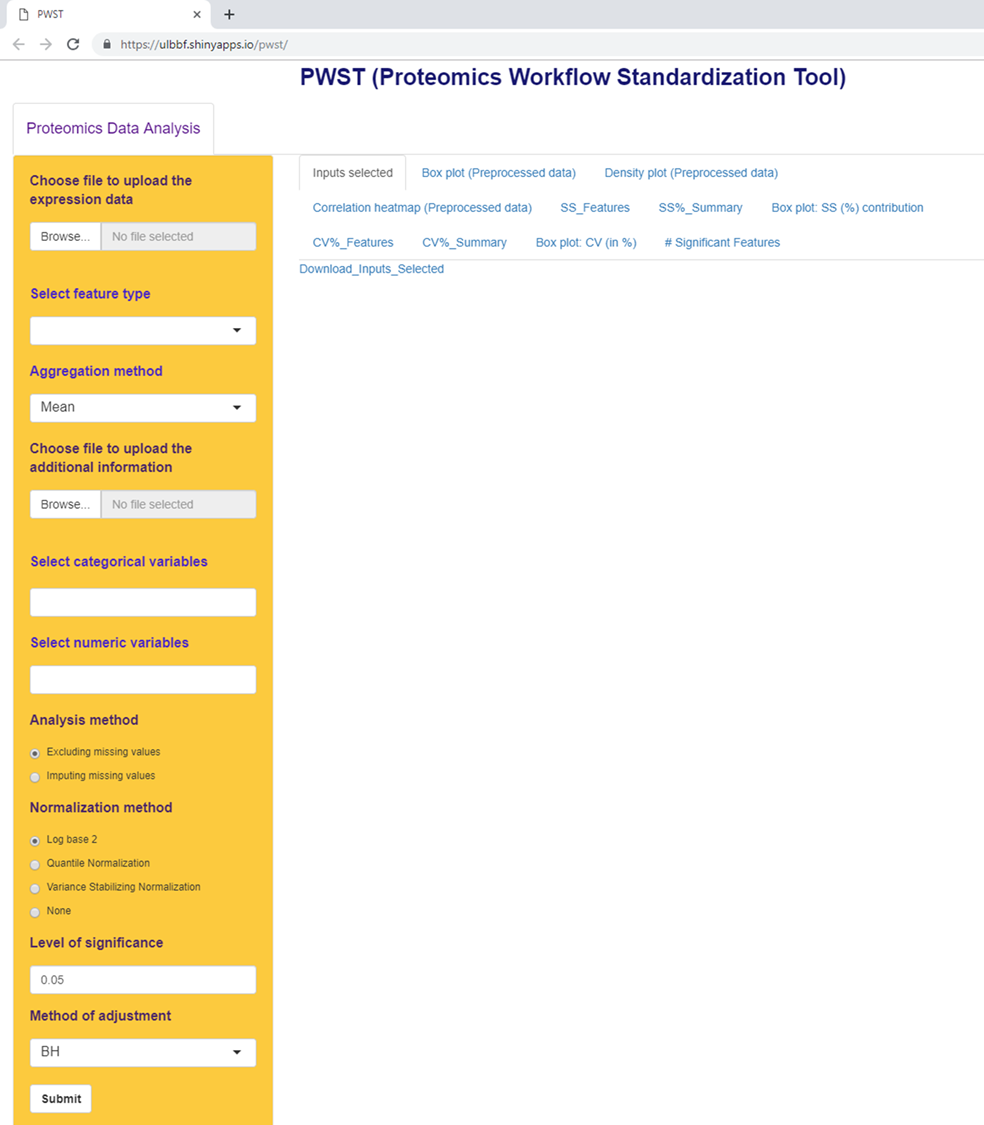


**Figure S1:** Webpage of the tool “PWST”

**Inputs to be specified by the user**

**1. Upload the expression data**

The user has to upload the proteomics expression data either in csv, txt, xls or xlsx format. The first two columns are reserved for proteins and peptides. Even if the data is available at protein level only and there is no peptide data, then user must leave the second column blank. The expression/abundance data must start from the third column and onwards. Therefore, the first row must contain the labels such as “Protein”, “Peptide” and the sample names (starting from third column). After the first row, we have the name of proteins and peptides in the first and second column respectively. In the remaining portion, we have the expression values of corresponding features (proteins and/or peptides) and samples. A portion of input expression data is shown below. Please see the example data file “Supplementary file 2.xlsx”.


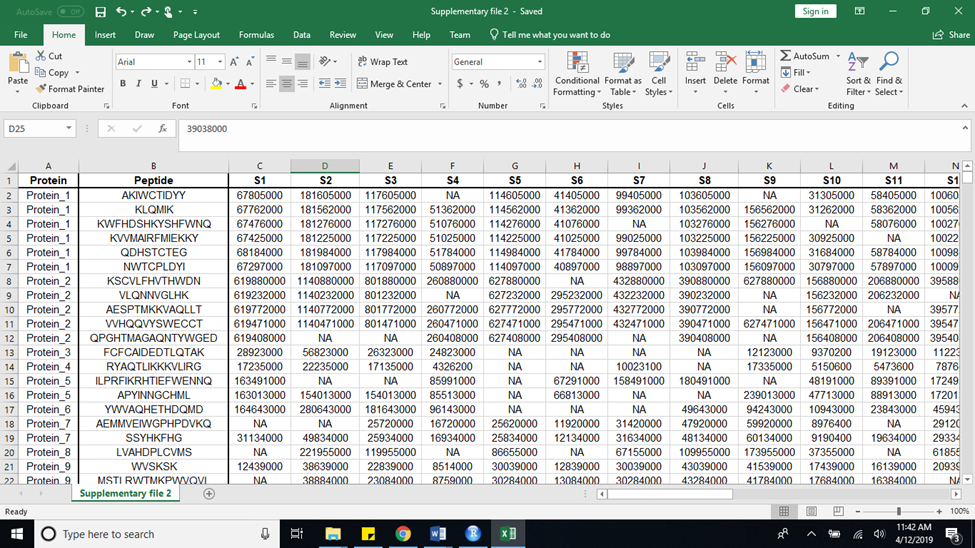


**Figure S2:** A portion of proteomics expression data

The user has to click on the “Browse…” button and select the file to upload the expression data as given below in Figures S3:


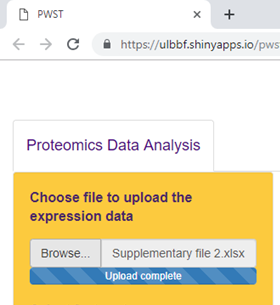


**Figure S3:** Upload the proteomics expression data

**2. Feature type**

After uploading the expression data file, the user has to select the feature type. The feature type available will automatically be detected. In the given example, we have the peptide data. So, there are two options available: “Protein” or “Peptide”. We selected the analysis to be done at “Protein” level as given below:


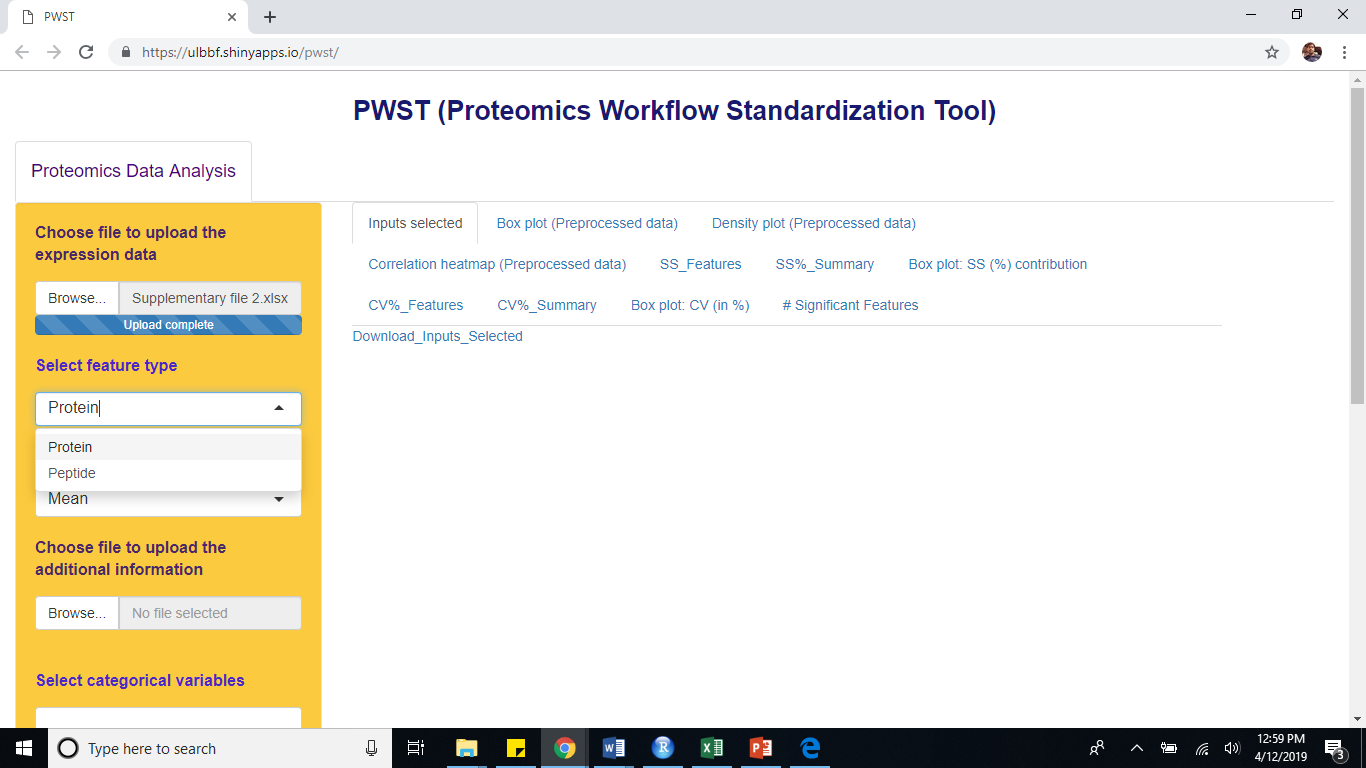


**Figure S4:** Choose the feature type – “Protein” or “Peptide”

**3. Aggregation method**

There are four options available for data aggregation: (i) Mean, (ii) Median, (iii) Sum, (iv) Maximum. We selected “Mean” for aggregating the peptide data at protein level as given below:


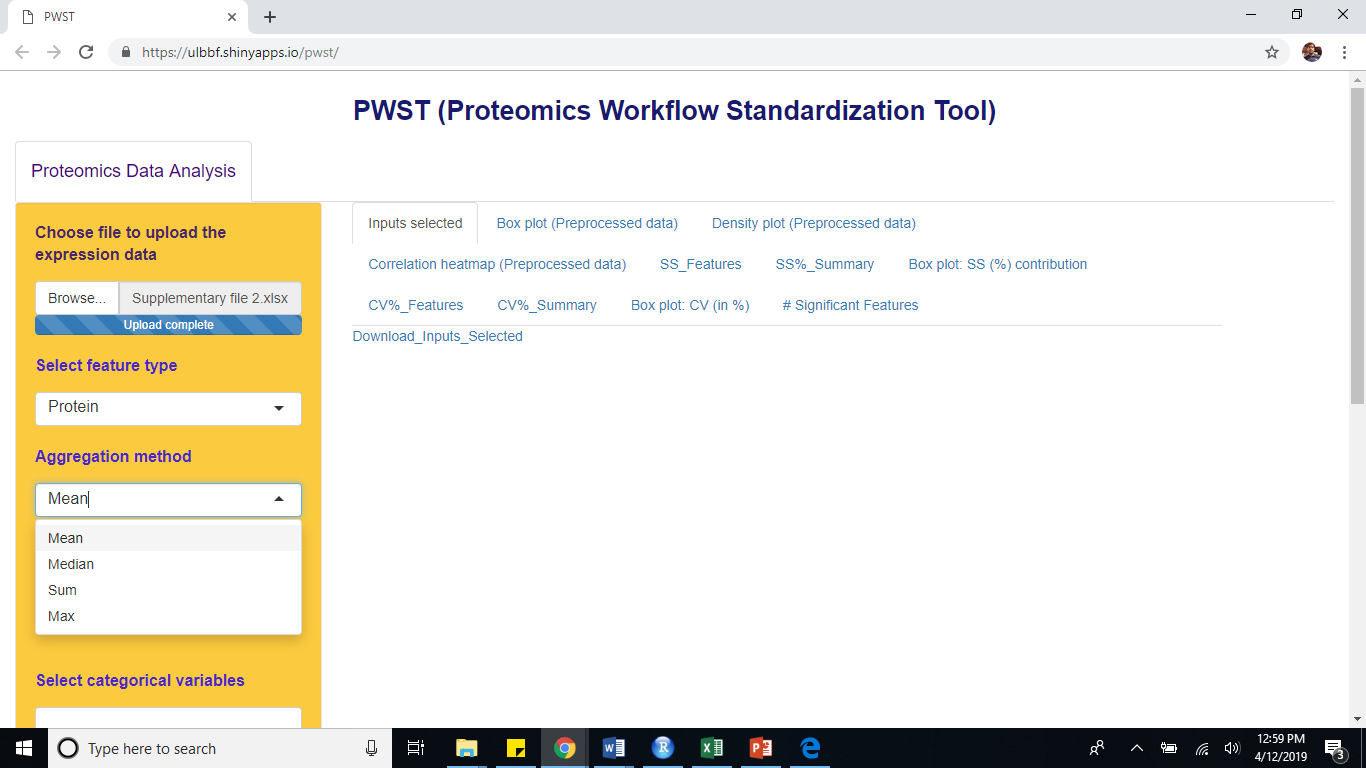


**Figure S5:** Choose the aggregation method (Mean/Median/Sum/Max)

**4. Upload the additional information**

Now the user has to upload the additional information about the data either in csv, txt, xls or xlsx format. This file contains the information of the samples and the variables under study. The variables may be categorical and/or continuous (numeric). A portion of additional data is shown below. Please see the example file “Supplementary file 3.xlsx” for complete information.


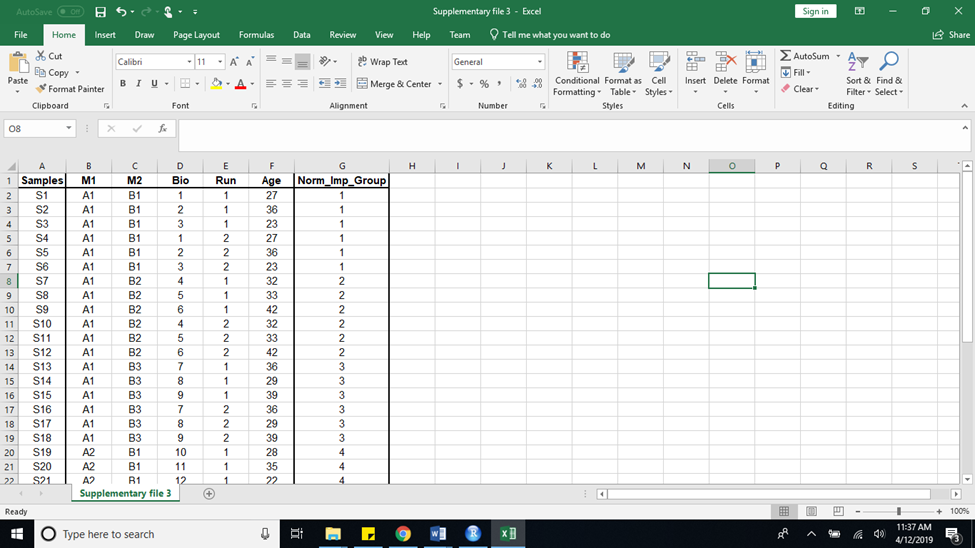


**Figure S6:** A portion of additional information of data

The user has to click on the “Browse…” button and select the file to upload the additional data as given below:


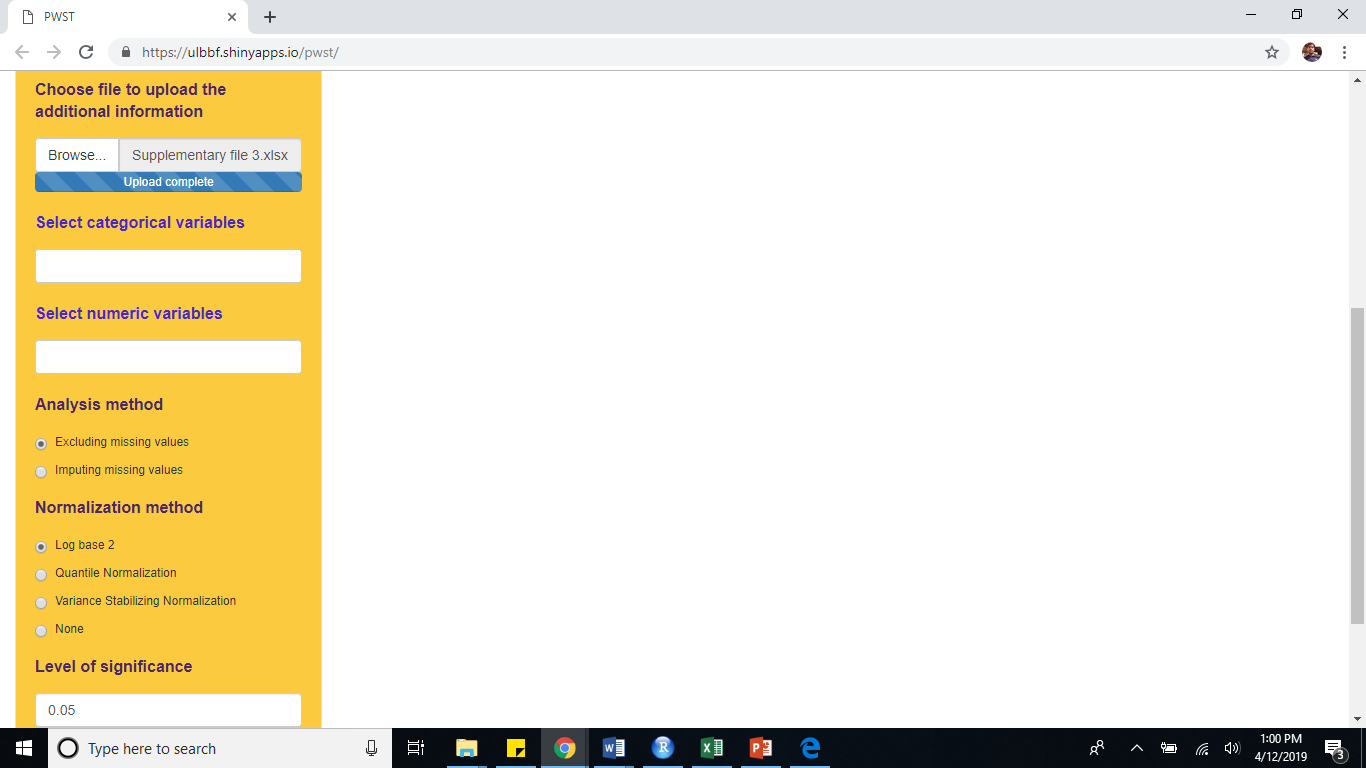


**Figure S7:** Upload the additional information of data

**5. Choose the categorical variables**

The user has to select the categorical variables one by one which will automatically pop out after the file containing additional information has been uploaded. We have selected “M1” and “M2” as the categorical variables under study as given below:


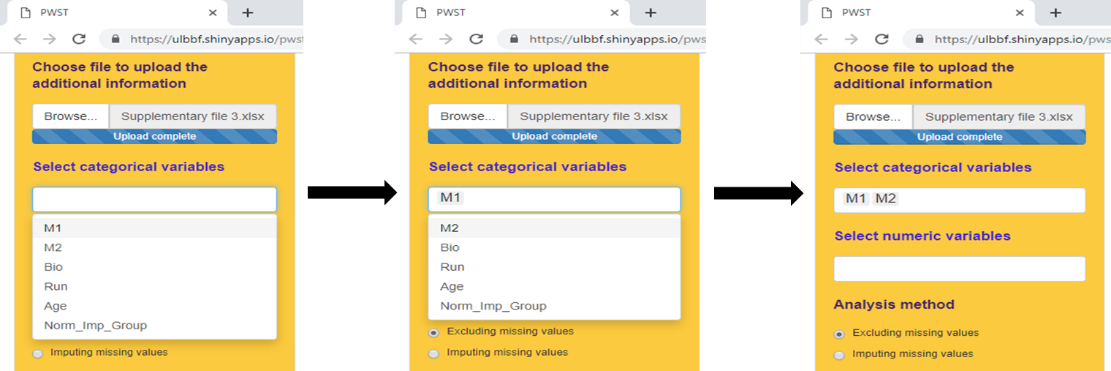


**Figure S8:** Selection of categorical variables

**6. Choosing the numeric variables**

After selecting the categorical variables, the user can now select the continuous variable from the remaining variables, if available. In this example, we have selected “Age” as given below:


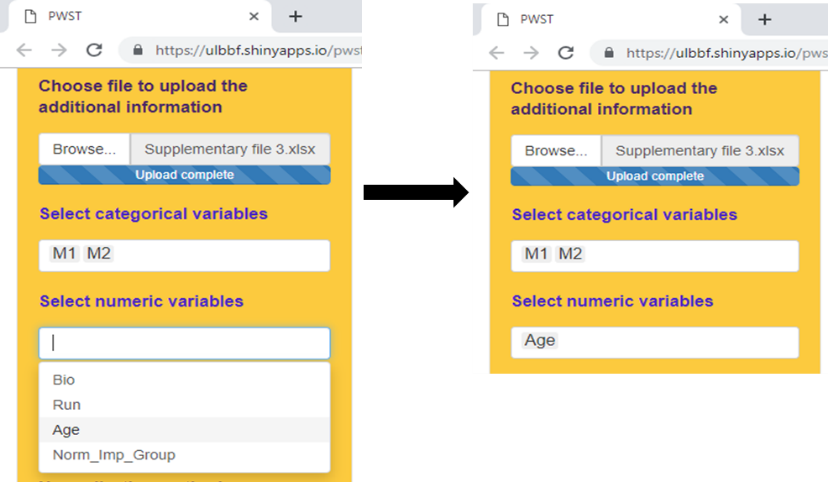


**Figure S9:** Selection of numeric variables

**7. Analysis method**

We have provided two options for the analysis: (i) Excluding missing values and (ii) Imputing missing values. Further, there are two methods of data imputation available: (a) SVD and (b) KNN. We selected the radio button “Imputing missing values” and “SVD” method for data imputation. The screenshots are given below:


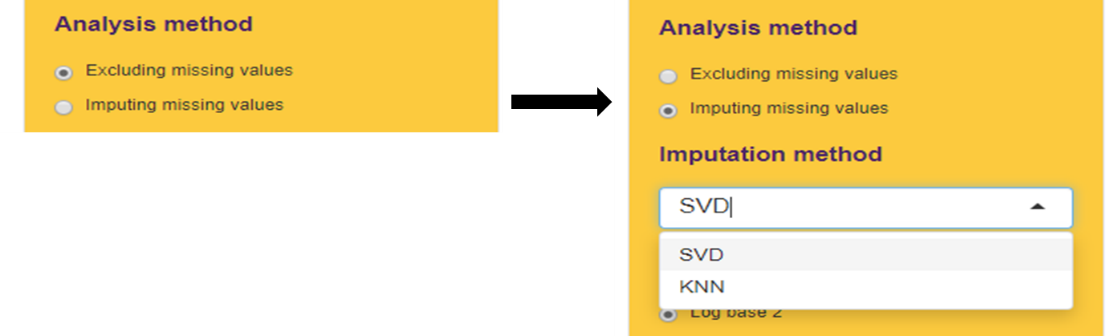


**Figure S10:** Selection of numeric variables

**8. Transformation/Normalization method**

There are four options available for data transformation and/or normalization. We selected “Variance Stabilizing Normalization” for data normalization as given below.


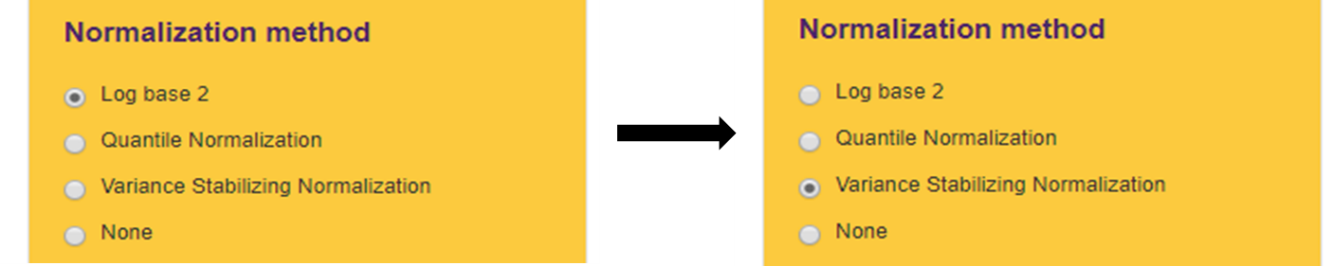


**Figure S11:** Selection of normalization method

**9. Level of significance**

The user has to specify the level of significance. We have selected the default value 0.05 as the level of significance.


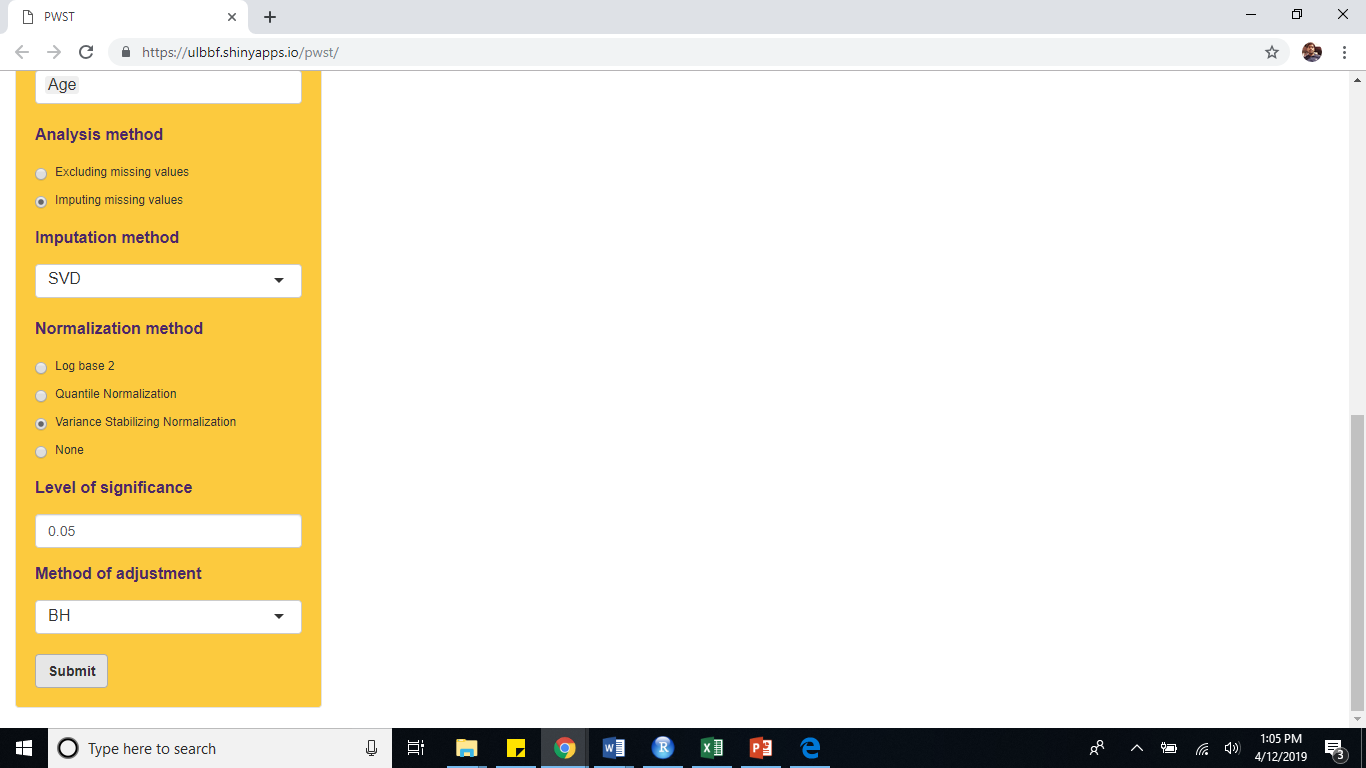


**Figure S12:** Specify the level of significance

**10. Method of adjustment**

The user has to select the method of adjusting the p-values for multiple testing of features. We have provided six adjustment methods. We selected “BH” adjustment method.

**
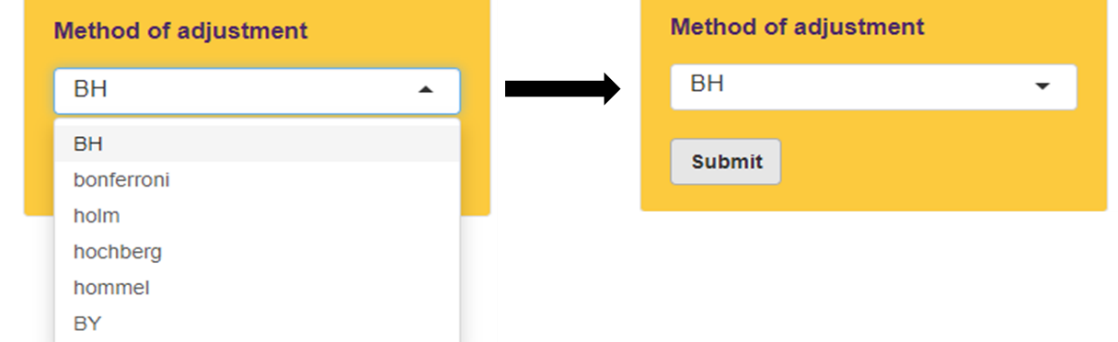
**

**Figure S13:** Specify the adjustment method

After specifying all the inputs, the user has to hit the “Submit” button and wait for the results.

**Results obtained**

**1. Inputs selected:** The various inputs defined by the user for the analysis can be viewed as given below.


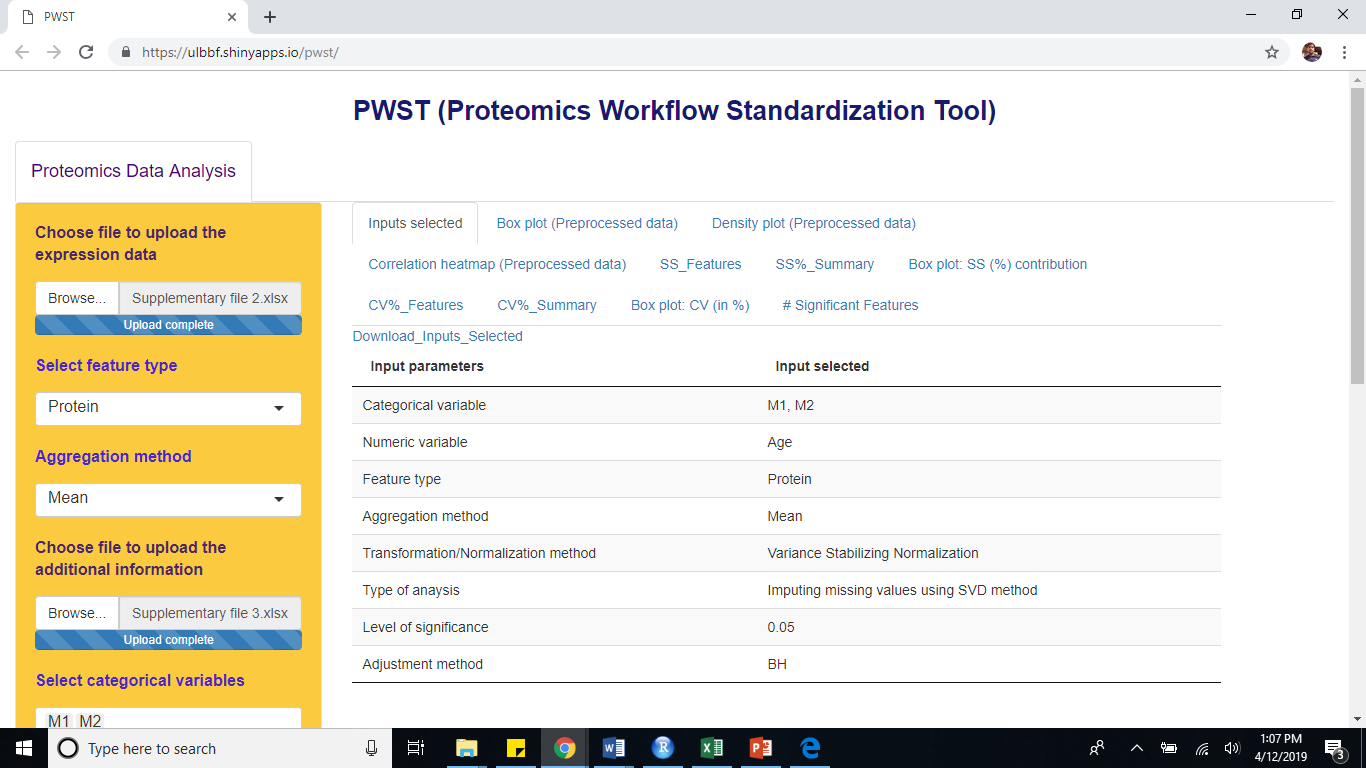


**Figure S14:** Inputs selected

**2. Visual plots of the preprocessed data:** The various exploratory plots of the preprocessed data such as box plot, density plot, correlation heatmap can be viewed under each tab as shown below:


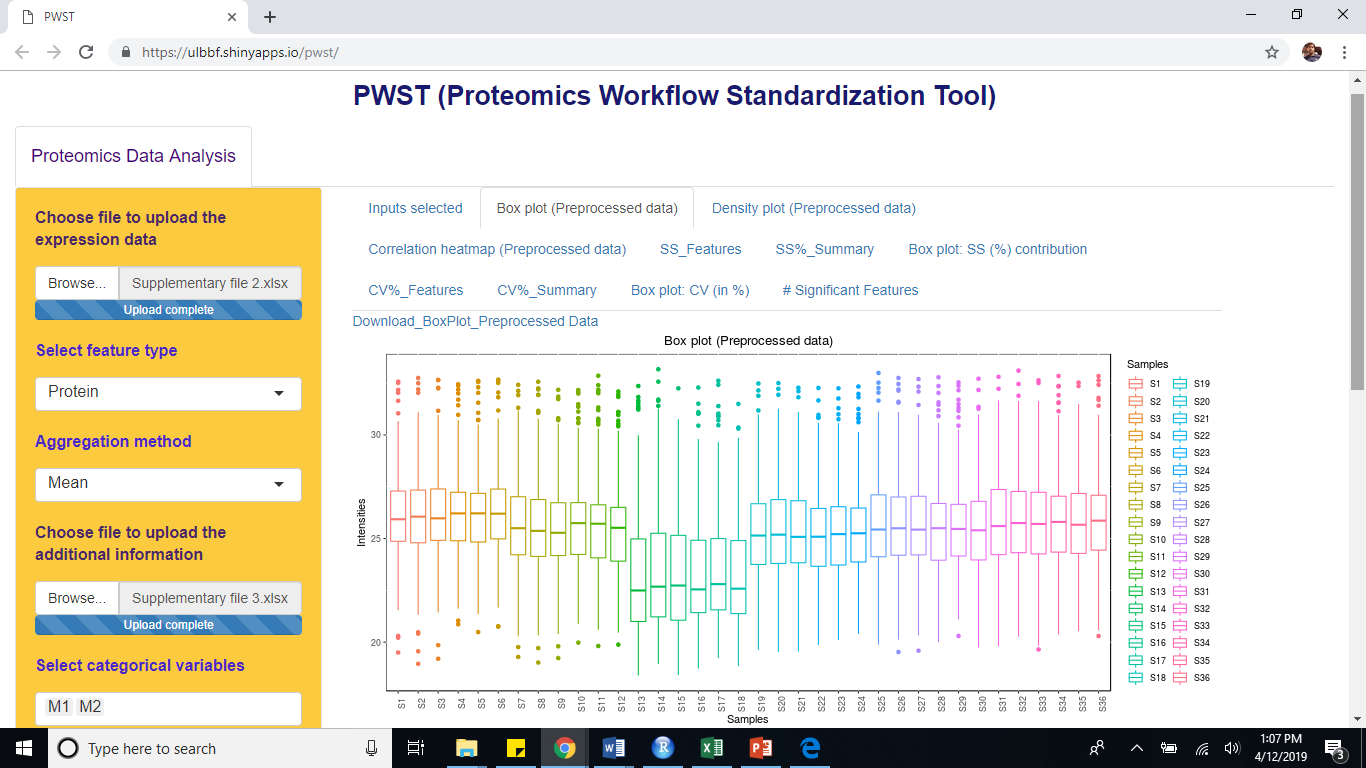


**Figure S15:** Box plot of preprocessed expression data


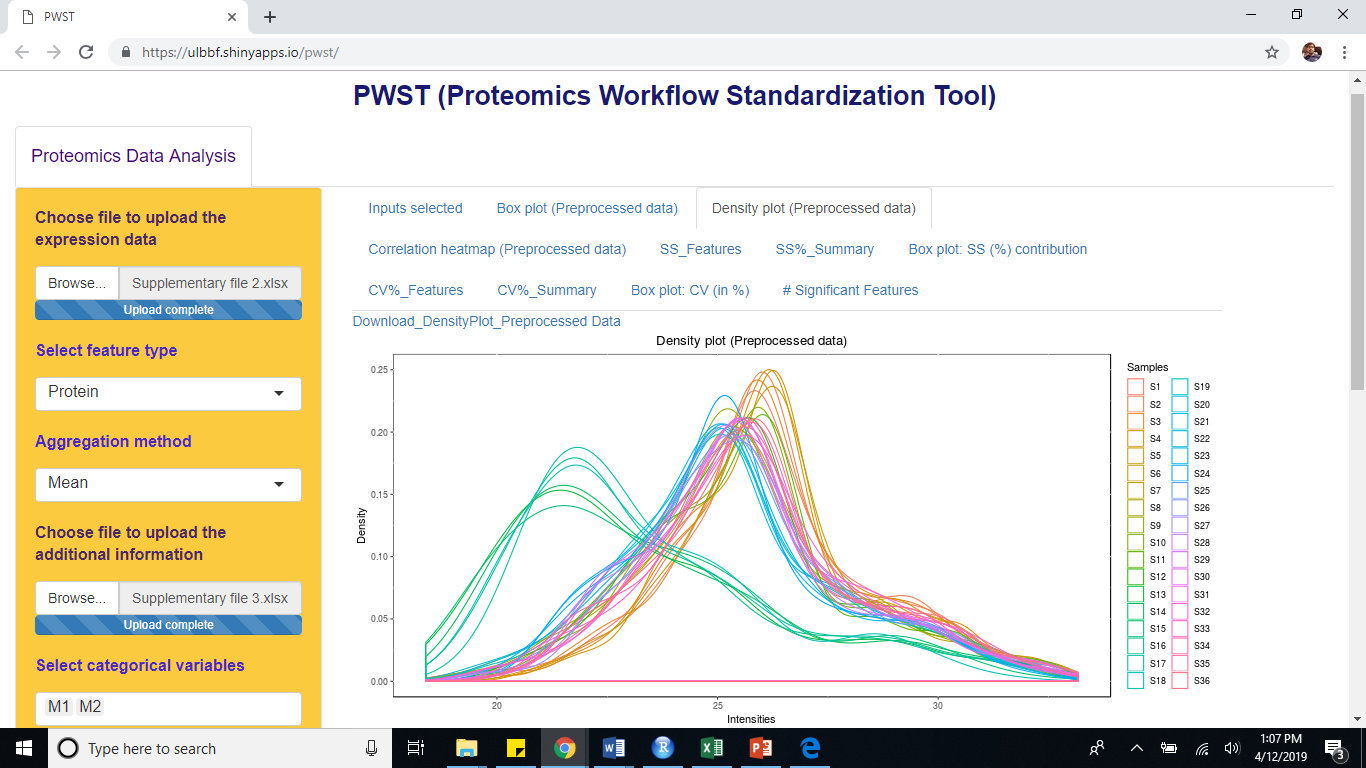


**Figure S16:** Density plot of preprocessed expression data


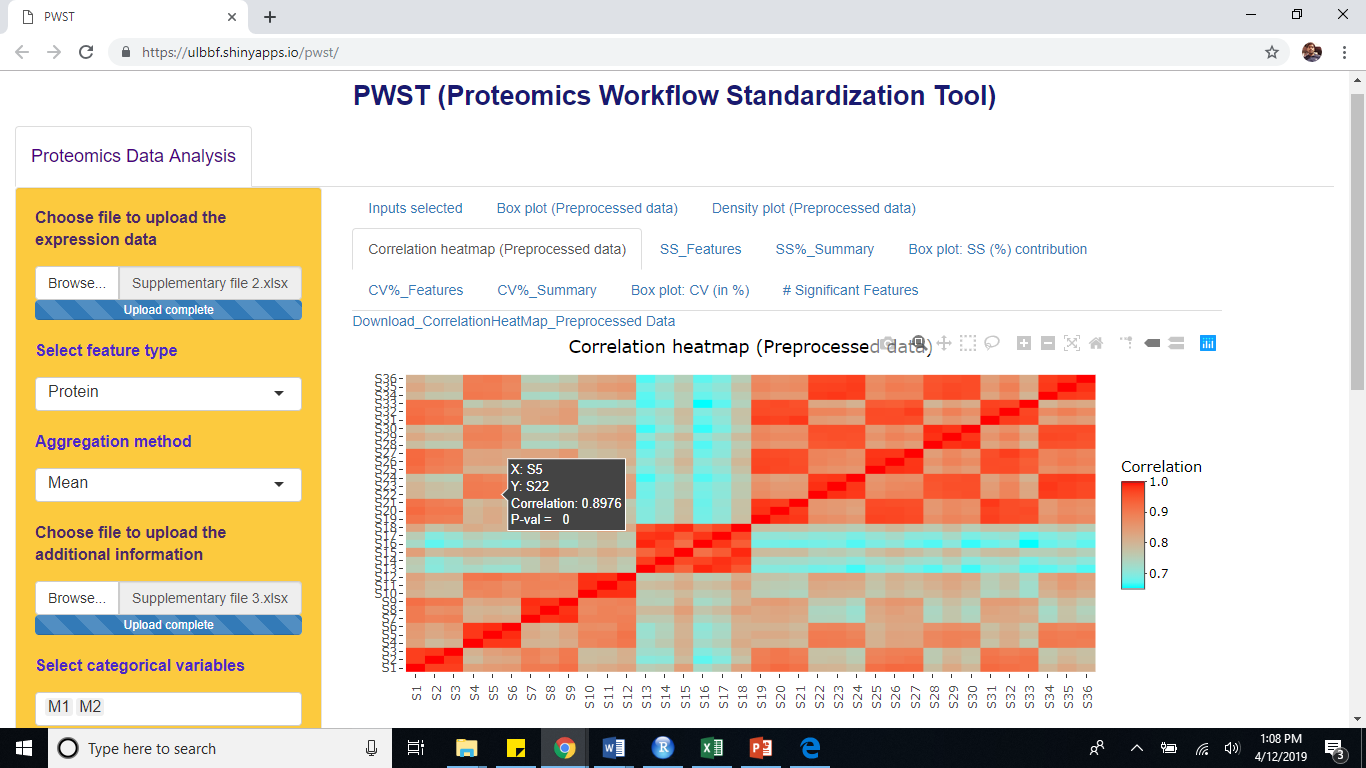


**Figure S17:** Interactive correlation heatmap of preprocessed expression data

From the box plot and density plot, we find that the data normalized using the “VSN” normalization method and analysis using “SVD” imputation method are normally distributed for all the samples.

The correlation heatmap shows correlation between the samples and the corresponding p-values.

**3. The SS results:** (i) The results showing the contribution of SS squares due to each variable, the p-values and the adjusted p-values corresponding to each variable are shown below. If the input is peptide data and analysis is at “Protein” level, the table will also show the number of peptides corresponding to each protein. The complete SS result can be downloaded by clicking on the “Download_Result_SS_Features” link.


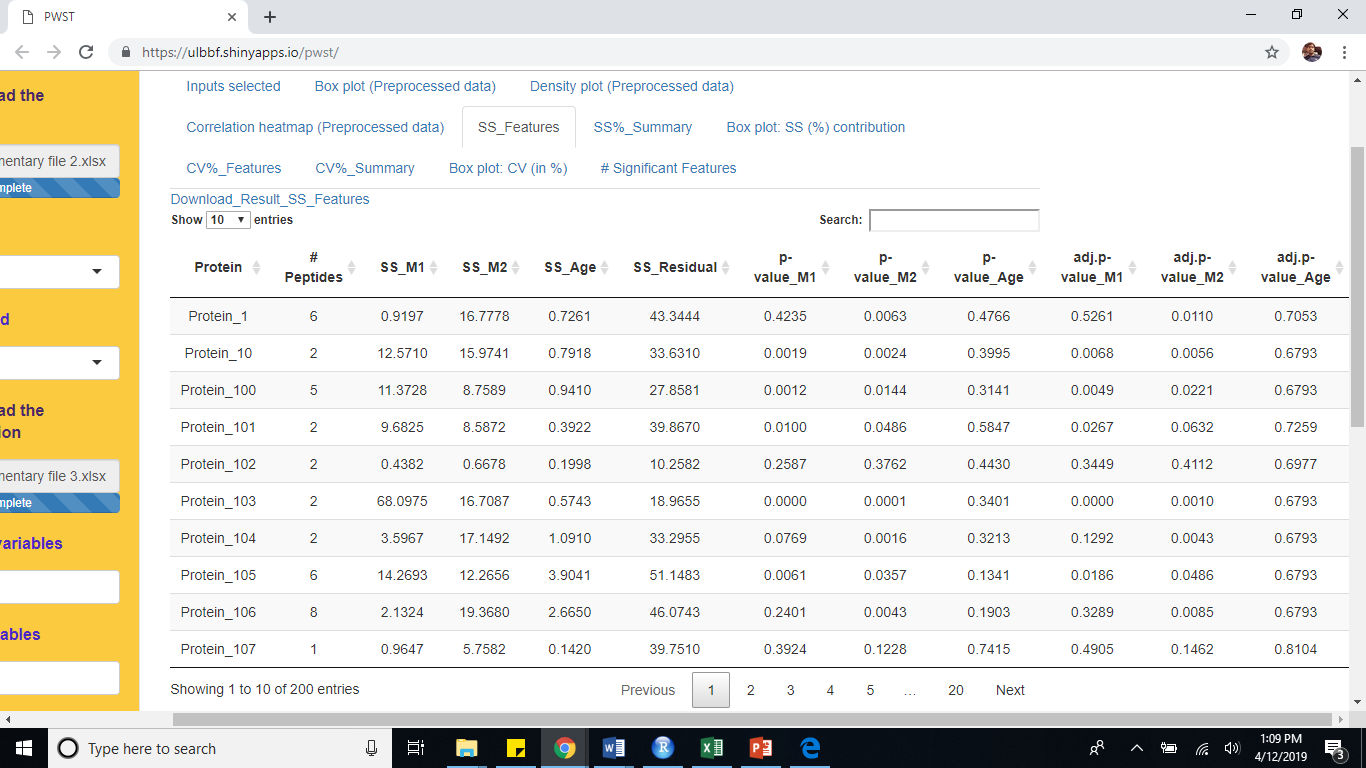


**Figure S18:** Contribution of SS due to each variable, the p-values and the adjusted p-values corresponding to each variable for each protein

(ii) The result summary of % SS contribution due to each variable is shown below:


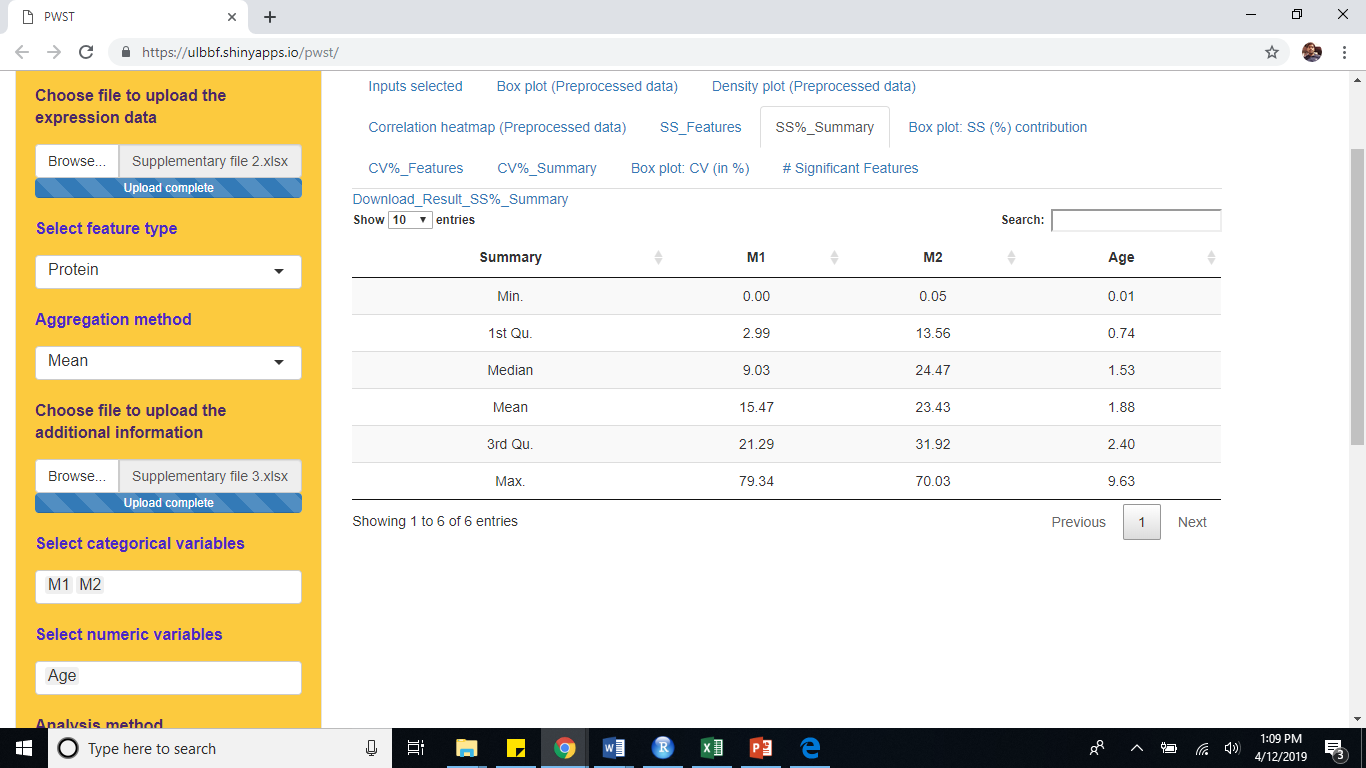


**Figure S19:** Summary of % SS contribution due to each variable

(iii) The box plot showing % contribution of SS due to each variable is shown below:


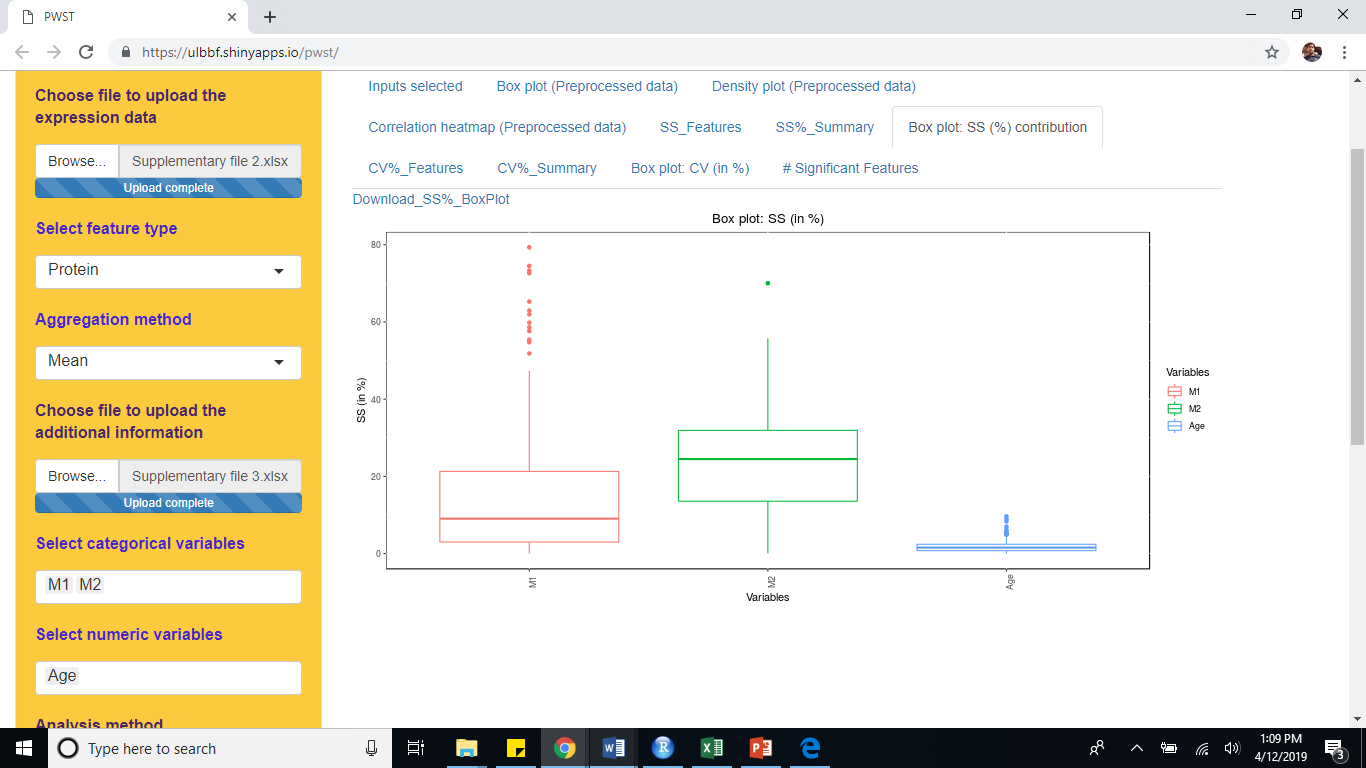


**Figure S20:** The box plot showing % contribution of SS due to each variable

From the summary and box plots, we found that the SS contribution due to the variable M2 is more than that of variable M1. The variable “Age” has the least SS contribution.

**4. The CV analysis:** We calculate the CV corresponding to the groups within each categorical variable. We obtained the following results: (i) CV of different groups of each categorical variable for all the proteins, (ii) Summary of CV (%) for all the proteins, and (iii) Box plot showing CV under the various groups of categorical variables. These results are shown below:


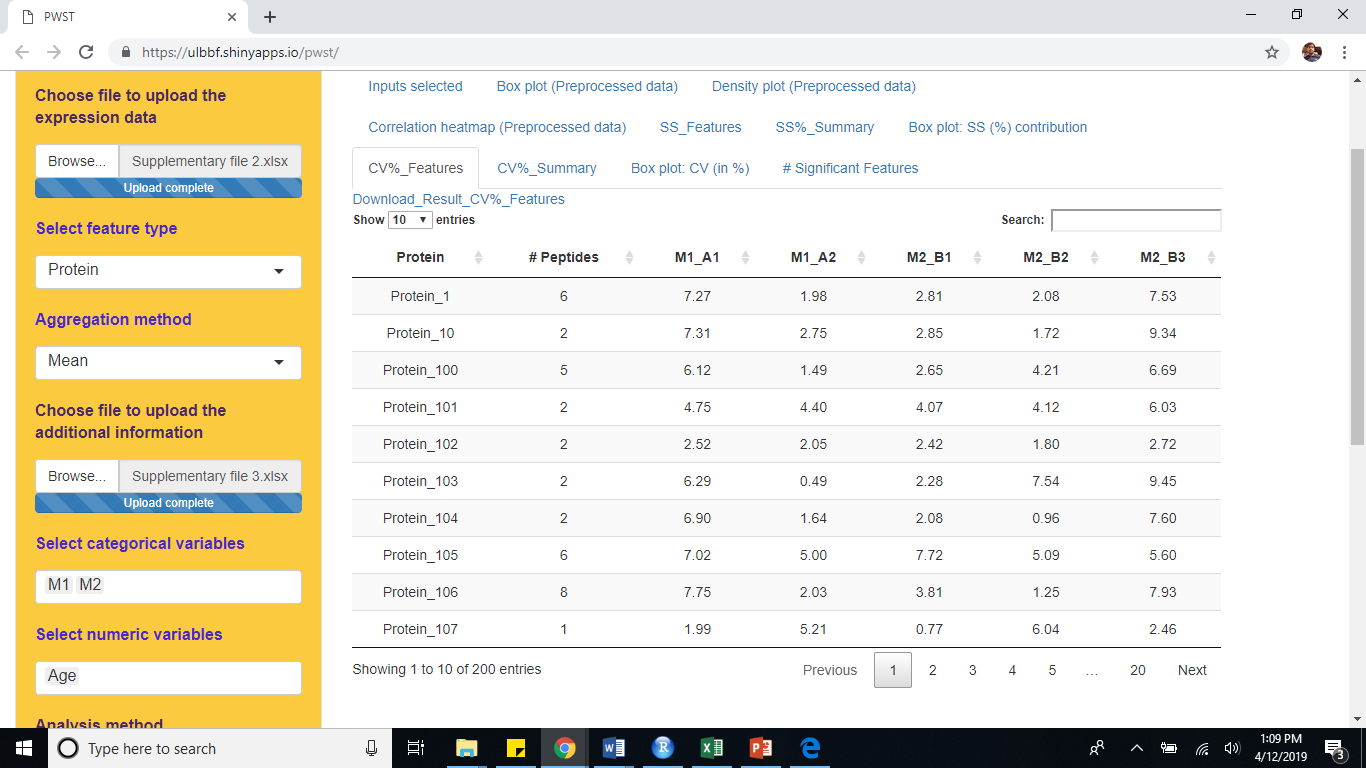


**Figure S21:** The CV of different groups of each categorical variable for all the proteins


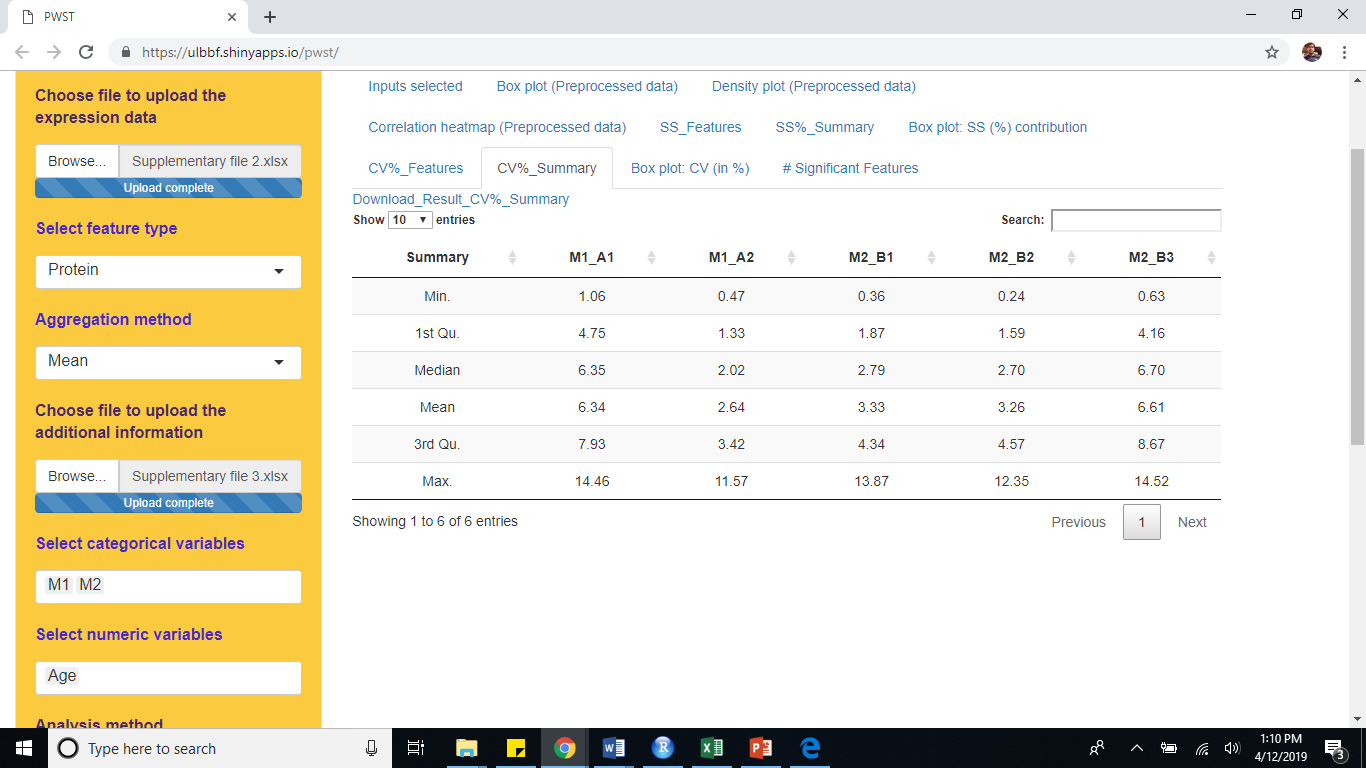


**Figure S22:** Summary of CV (%) for all the proteins


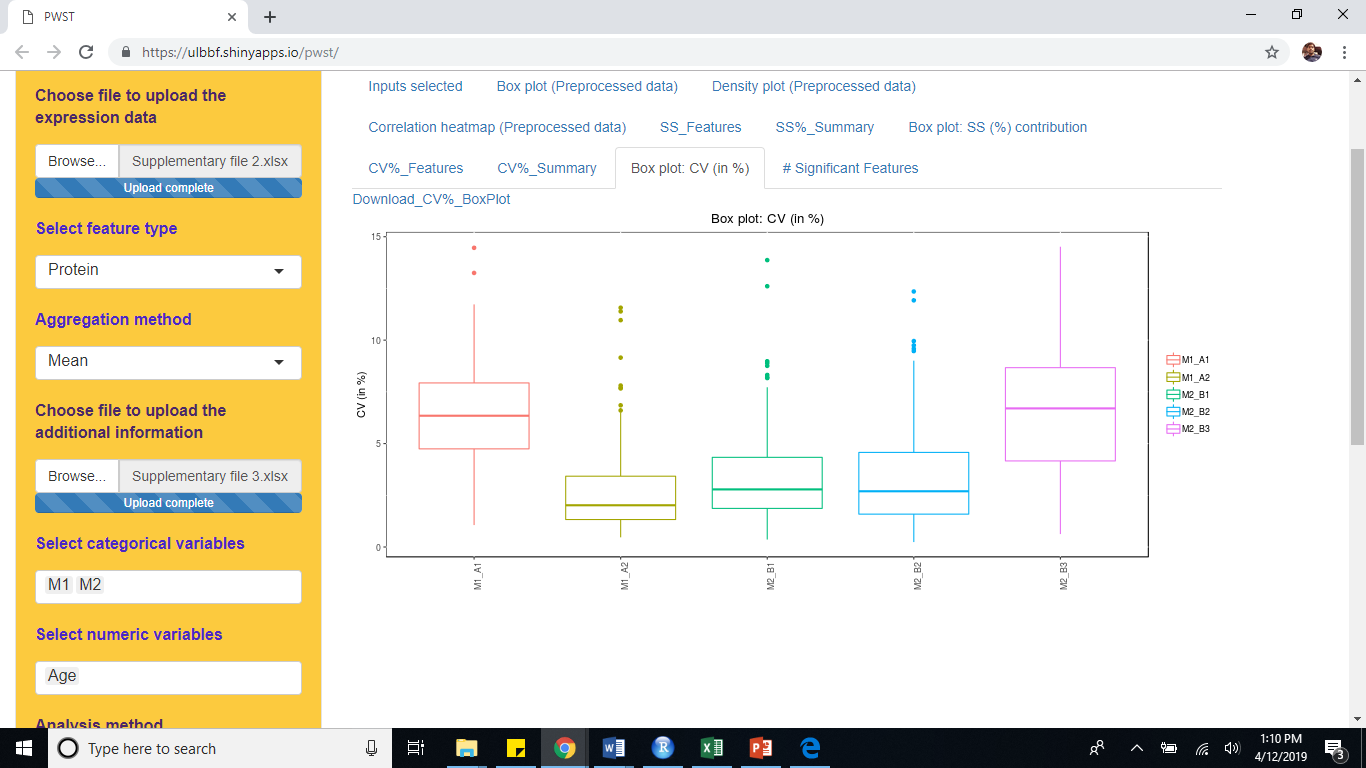


**Figure S23:** Box plot showing CV under the various groups of categorical variables

The summary and box plots of CV show that (i) within variable M1, A2 has lesser variability that of A1 and (ii) within variable M2, B2 has the least variability among the three approaches of M2.

**5. Number of significant features:** A table showing the total number of proteins assessed and the number of proteins which have significant effect due to each variable, “M1”, “M2” and “Age”, without and with adjustment is shown below.


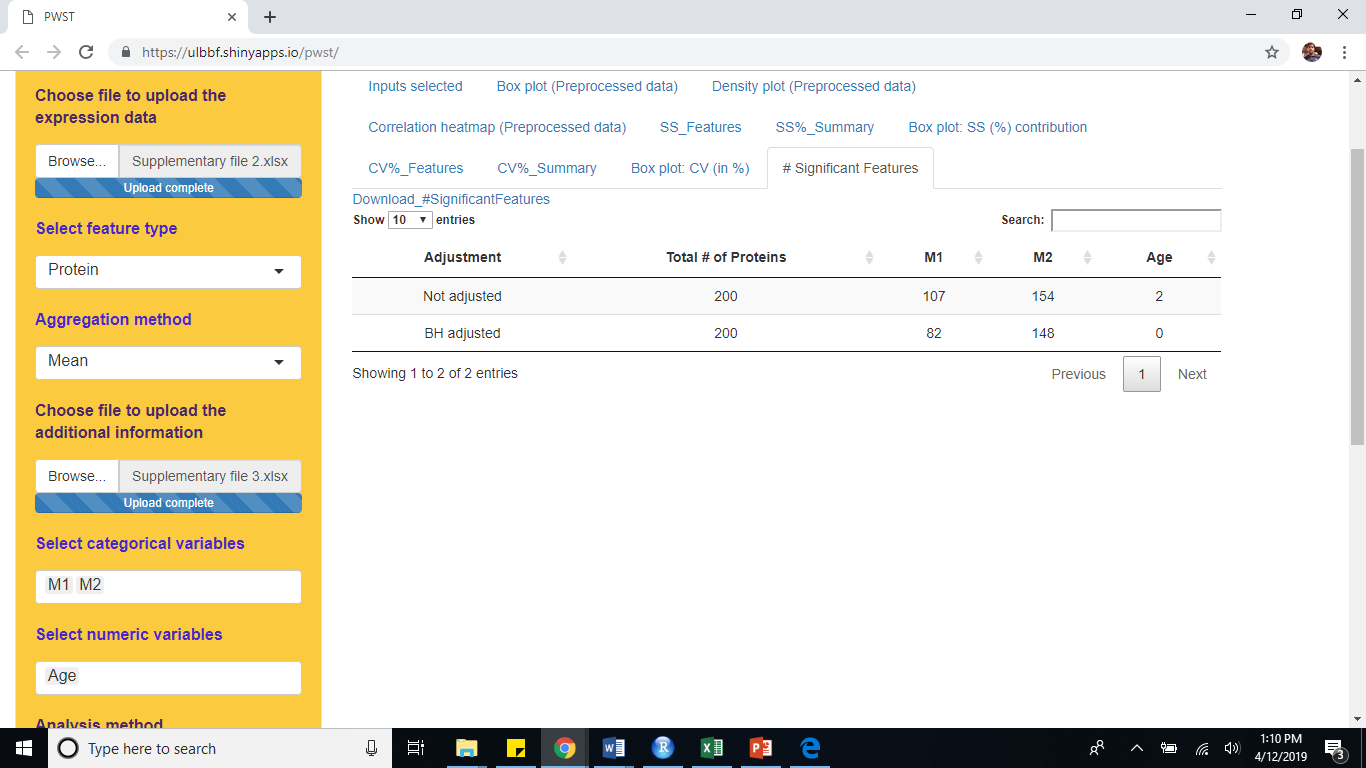


**Figure S24:** Summary of significant proteins

We found greater number of proteins showing significant effects due to M1 and M2. This further shows that variable M2 has more significant effect than that of M1. The variable “Age” has very less effect, i.e., only two protein showed significant effect due to “Age”.

The user can download the results under each tab by clicking on the download links provided under each tab. The tables will be downloaded in “xlsx” format and the plots will be download in “png” format.
